# Supplementary material for: Rapid turnover of life-cycle-related genes in the brown algae
Source: Genome Biol. 2019 Feb 14;20:35. doi: 10.1186/s13059-019-1630-6 (PMC6374913; doi:10.1186/s13059-019-1630-6)
Supplement: Supplementary file 3 — Protein alignments, corresponding Gblocks html file and CDS sequences for the lineage conserved gametophyte-biased genes. (ZIP 2109 kb) [file 13059_2019_1630_MOESM3_ESM.zip › Con_GA_alignments/Ec-14_003300.aa_ali.fasta-gb.htm]

Ec-14\_003300.aa.fasta.trans.aa\_ali.fasta


## Gblocks 0.91b Results

Processed file: **Ec-14\_003300.aa.fasta.trans.aa\_ali.fasta**  
Number of sequences: **2**  
Alignment assumed to be: **Protein**  
New number of positions: **195** (selected positions are underlined in blue)

```
                         10        20        30        40        50        60
                 =========+=========+=========+=========+=========+=========+
Ec-14_003300     MDTTTEEGSDSPAGGEVREEQEFLRWLRSHGAAIDCVEWPSSETESGVRGAVARRDIAPG
MSTRG.41192.1    MDKSAE-GEGSNAEPAAMAEQQFLDWLRRHGAGIDSIDWPSSETGSGIRGAVAKQDIEGG
                        #####################################################


                         70        80        90       100       110       120
                 =========+=========+=========+=========+=========+=========+
Ec-14_003300     DHMVIIPHALMMSEFHAKADPKYGHVHRLNTRLLGSDNGLALYIMQEILKEERSFYWPYL
MSTRG.41192.1    EHMVVIPRDLMMSESHAKADPQFGHVHRLHDRLLGSDNGLAVYVMQEILKGDRSFYRPYL
                 ############################################################


                        130       140       150       160       170       180
                 =========+=========+=========+=========+=========+=========+
Ec-14_003300     RMLPTPCNLRNWNRESLLLLQDHKLVRRTAARSRQLLALYRETIEFLSSSYPELYTADRY
MSTRG.41192.1    RVLPTPRNLQNWDEEDLLPLQDRKLVRRTASRKRQLQALYQETIGFLSSNYPDLYAAERY
                 ############################################################


                        190       200       210       220       230       240
                 =========+=========+=========+=========+=========+=========+
Ec-14_003300     TFELFDFAWRTIQARAFGKRLKSSALVPFADCLNHGNVQTKYDFDVGGNGTFRLFPSGNN
MSTRG.41192.1    TFELFAFAWSTVQARAFGKRLK--------------------------------------
                 ######################                                      


                        250       260       270       280       290       300
                 =========+=========+=========+=========+=========+=========+
Ec-14_003300     RYPRNSEVLNSYGRRANDNLLLDYGFAMLDNEWDAAEVICSLPPSHDQSPLDRRRKACLR
MSTRG.41192.1    ------------------------------------------------------------
                                                                             


                        310       320       330       340       350       360
                 =========+=========+=========+=========+=========+=========+
Ec-14_003300     ASGQHTVRILRVRRDVWPEELLRFCRCACLTAPELDNLEALGGQQARMASQSRPSGAMRP
MSTRG.41192.1    ------------------------------------------------------------
                                                                             


                        370       380       390       400       410       420
                 =========+=========+=========+=========+=========+=========+
Ec-14_003300     RGIAGTVEPTAAAVPPYQDLCRCARRSSKQATRGRAECGSGSGGSGDRTATGGATAAPLS
MSTRG.41192.1    ------------------------------------------------------------
                                                                             


                        430       440       450       460       470       480
                 =========+=========+=========+=========+=========+=========+
Ec-14_003300     SSLPSCEKAREDTINNNNGDDDTLREGLSVHASKCTLPLRDEHEEGSGDRGDRGAGAGAK
MSTRG.41192.1    ------------------------------------------------------------
                                                                             


                        490       500       510       520       530       540
                 =========+=========+=========+=========+=========+=========+
Ec-14_003300     EEGQEDGDVPSGAGGAGEKRGGVDSPDENPDASGCRFPCRALTAQNEVAALTEAVKYLAQ
MSTRG.41192.1    ------------------------------------------------------------
                                                                             


                        550       560       570       580       590       600
                 =========+=========+=========+=========+=========+=========+
Ec-14_003300     ARDNYPTTLEHDENAYREARTSPDSSPELATALIYRVTVKRILARQLEMAEAANAFVQNI
MSTRG.41192.1    ------------------------------------------------------------
                                                                             


                        610       620       630       640       650       660
                 =========+=========+=========+=========+=========+=========+
Ec-14_003300     AAVRDQFQSSDENGKGAGHENGSSMVSAPVSSSRLDHLLASPPSPPSRLSSPPLSTRASS
MSTRG.41192.1    ------------------------------------------------------------
                                                                             


                        670       680       690       700       710
                 =========+=========+=========+=========+=========+=========
Ec-14_003300     TTGGGIENKCHQHHLQSHRFSLATGIDSPSFVSYLADVESVIVYQREAATAAAAAYQPE
MSTRG.41192.1    -----------------------------------------------------------
```

```
Parameters used
Minimum Number Of Sequences For A Conserved Position: 2
Minimum Number Of Sequences For A Flanking Position: 2
Maximum Number Of Contiguous Nonconserved Positions: 8
Minimum Length Of A Block: 10
Allowed Gap Positions: None
Use Similarity Matrices: Yes
```

```
Flank positions of the 1 selected block(s)
Flanks: [8  202]  

New number of positions in Ec-14_003300.aa.fasta.trans.aa_ali.fasta-gb:  195  (27% of the original 719 positions)
```
